# Clinical Outcomes of Single Mosaic Embryo Transfer: High-Level or Low-Level Mosaic Embryo, Does It Matter?

**DOI:** 10.3390/jcm9061695

**Published:** 2020-06-02

**Authors:** Pin-Yao Lin, Chun-I Lee, En-Hui Cheng, Chun-Chia Huang, Tsung-Hsien Lee, Hui-Hsin Shih, Yi-Ping Pai, Yi-Chun Chen, Maw-Sheng Lee

**Affiliations:** 1 Institute of Medicine, Chung Shan Medical University, No. 110, Sec. 1, Jianguo N. Rd., South District, Taichung City 40201, Taiwan; jellylin0607@gmail.com (P.-Y.L.); adoctor0402@gmail.com (C.-I.L.); jackth.lee@gmail.com (T.-H.L.); 2 Division of Infertility, Lee Women’s Hospital, No. 30-6, Sec. 1, Changping Road, Beitun District, Taichung City 406, Taiwan; enhuicheng@gmail.com (E.-H.C.); agarhuang@gmail.com (C.-C.H.); chloroplast7668@gmail.com (H.-H.S.); dancing_girl0926@yahoo.com.tw (Y.-P.P.); alice4918430@gmail.com (Y.-C.C.); 3 Department of Obstetrics and Gynecology, Chung Shan Medical University Hospital, No. 110, Sec. 1, Jianguo N. Rd., South District, Taichung City 40201, Taiwan

**Keywords:** in vitro fertilization, preimplantation genetic testing for aneuploidy, aneuploidy, mosaic embryo, next-generation sequencing

## Abstract

Recently, reports showed that embryos identified as mosaic after preimplantation genetic testing for aneuploid (PGT-A) could result in live birth with lower pregnancy and higher pregnancy loss rates compared with euploid embryos. However, the effects of mosaicism level on reproductive outcomes remain controversial. This study aimed to examine the level of mosaicism on pregnancy outcomes. Single mosaic embryo transfer was offered to 108 women who only had mosaic embryos. Mosaic embryos were labeled by utilizing next generation sequencing (NGS) based PGT-A for day 5/6 trophectoderm (TE) biopsies. TE biopsies containing < 50% abnormal cells were classified as low-level mosaicism and ≥ 50% as high-level mosaicism. To further confirm the concordance of chromosome constitution between TE and inner cell mass (ICM), 41 remaining embryos designated as mosaic blastocysts donated for research were also analyzed. Comparable live birth rate (LBR) but higher miscarriage rate (MR) was found in the high-level group. (LBR: low vs. high: 44.5% vs. 36%; *p* = 0.45, MR: low vs. high: 5.1% vs. 30.7%; *p* = 0.012). Analyses of TE and ICM from the remaining mosaic blastocysts show a poor concordance. This preliminary study demonstrated that high-level mosaic embryos could result in comparable LBR but higher MR.

## 1. Introduction

Preimplantation genetic testing for aneuploidy (PGT-A) aims to improve in vitro fertilization (IVF) outcomes by avoiding aneuploidy embryo transfer. Compared with other methods for PGT-A, higher-resolution next-generation sequencing (hr-NGS) has higher chromosomal analysis resolution and is capable of detecting mosaicism levels as low as 20% [1]. Mosaic embryos are defined as the presence of two or more karyotypically distinct cell lines within the same embryo [2,3]. With increasing use of hr-NGS as the platform of PGT-A, more embryos are labeled as mosaic, which leads to many challenging clinical questions for both clinicians and patients, not only regarding the causes of mosaicism but also its clinical outcome. Recently published data suggest that mosaic embryos result in a live birth, but with a lower pregnancy rate and a higher pregnancy loss rate compared with euploidy embryos [4,5,6,7,8,9,10]. However, considerable uncertainty remains regarding the relation with clinical outcomes, type and level of mosaicism, and the number of chromosomes involved, and clarification of these issues is needed before prioritizing mosaic embryos. Current follow-up data obtained after mosaic embryo transfer is still insufficient to draw any conclusion related to which mosaicism should be prioritized for transfer.

Mechanisms of mosaic embryo formation include anaphase lag, mitotic errors, inadvertent chromosome demolition, and premature cell division before DNA duplication [3,11]. Mosaicism levels depend on the timing of mitotic errors, and more aneuploidy cells in the mosaic embryo presumably have a detrimental effect on embryo viability. Our previous study [12] used a time-lapse (TL) culture system and found that high-level mosaic embryos exhibit features related to mitotic errors and delayed development at the 8-cell stage. However, the effects of mosaicism levels on reproductive outcomes remain controversial. Spinella et al. [8] reported that mosaicism levels influence the IVF success rate, but others failed to find this correlation [7,13].

Therefore, we conducted a retrospective study to assess the effects of mosaicism on IVF outcomes after a single mosaic embryo transfer. This study provides new information on the role of mosaicism on the prioritization of mosaic embryo selection.

## 2. Material and Methods

A total 1162 NGS-based PGT-A cycles were performed at the Lee Women’s Hospital, Taiwan, from July 2016 through July 2018. The indications for PGT-A were recurrent IVF failure (39.7%), advanced maternal age (49.4%), recurrent miscarriage (13.2%), male factor (4.8%), combined preimplantation diagnosis (7.2%) and unexplained infertility (7.2%). Some couples may have had more than one indication for PGT-A.

**Figure 1:**
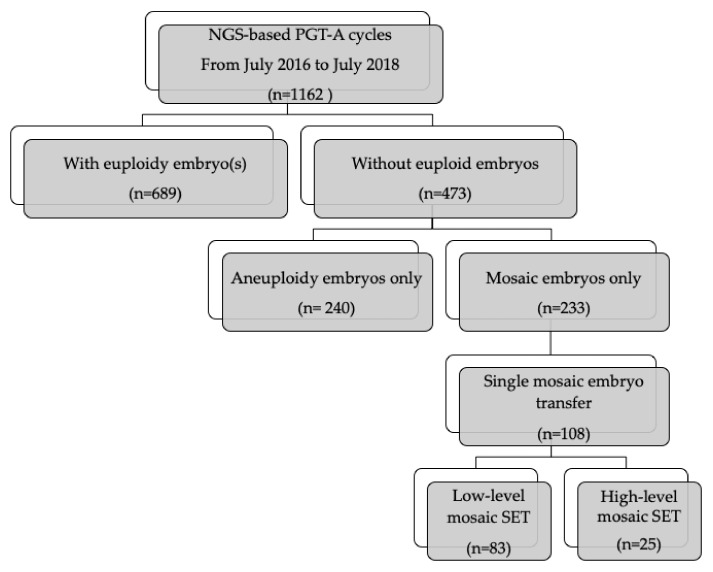
Flowchart of study population.

Among the 1162 PGT-A cycles, 233 (20.1%) reported only mosaic embryos and 240 (20.7%) only aneuploid embryos. Utilizing NGS, embryos were determined as having mosaicism when a whole chromosome or sub-chromosomal segment showed intermediate copy number levels (in the range of 20%–80% between whole numbers), following Preimplantation Genetic Diagnosis International Society (PGDIS) guidelines [1,6]. Low-level mosaicism was defined as less than 50% and high-level mosaicism as equal or higher than 50% (Figure 1).

## 3. Study Population

This retrospective, single-center, cohort study included patients who had no euploid embryo after PGT-A. After counseling and informed consent, 108 patients underwent single embryo transfer (SET) with mosaic embryos at the Lee Women’s Hospital, Taiwan, from July of 2016 to July of 2018. SET cycles of 83 low-level and 25 high-level mosaic transfers were retrospectively analyzed. Egg donation cycles and repeat PGT-A cycles were excluded. If pregnancy continues to a gestational age of 16 to 18 weeks, amniocentesis for karyotyping would be performed for all women. Prenatal ultrasound examinations for targeted anatomical evaluations were performed during 20–24 weeks with close monitoring of pregnancy across all trimesters. The study protocol was approved by the Institutional Review Board (IRB) of Chung Shan Medical University Hospital (IRB: CS19039), and we obtained written informed consent from each woman before proceeding with mosaic embryo transfer.

To confirm the concordance of chromosome constitution between trophectoderm (TE) and inner cell mass (ICM), 41 remaining mosaic blastocysts were donated for research after obtaining informed consent. This study was approved by Chung Shan Medical University Hospital (IRB: CS16105).

## 4. In Vitro Fertilization, Embryo Culture, TE Biopsy, and Frozen Embryo Transfer

The protocols for ovarian stimulation were described previously [12]. All patients underwent controlled ovarian hyperstimulation treatment using the gonadotropin-releasing hormone (GnRH) agonist long protocol or the GnRH antagonist protocol. Oocyte retrieval, micromanipulation, embryo culture, TE biopsy, and embryo vitrification or warming were performed following our standard protocols [14]. On day 5 or 6, the blastocysts were graded according to the Gardner Blastocyst Morphologic Scoring System. The quality of expanded blastocysts on day 5 or 6 was graded immediately before TE biopsy. According to Gardner grading system criteria, the expanded blastocysts with at least moderate quality of ICM and TE (i.e., AA, BA, CA, AB, BB, CB, AC, and BC) were considered of good quality. Approximately 5–10 TE cells were biopsied from good blastocysts on day 5 or 6 using laser-assisted hatching, after which the blastocysts were vitrified. All patients underwent frozen embryo transfer with either a natural or an artificial cycle for endometrial preparation using estradiol and progesterone supplementation. An endometrial thickness of at least 8 mm must be achieved before FET.

## 5. PGT-A Using hr-NGS

We used the Veriseq NGS (Illumina, San Diego, CA, USA) platform. Validation of mosaicism with mixing experiments to determine mosaicism levels in TE biopsies and define the proper thresholds to discriminate levels of mosaicism has been previously performed [6,15]. We conducted proof-of-principle mixing experiments to evaluate the sensitivity of the Illumina Veriseq NGS platform in mosaicism detection. Normal embryonic stem cell lines (46XY) and aneuploid cell lines that included trisomy of chromosome 16 (47XY+16) were mixed in different ratios, creating a mixture of six cells with different proportions of abnormal alleles of interest (0%, 20%, 30%, 40%, 50%, 60%, 70%, 80%, and 100%).

According to the manufacturer’s protocol, genomic DNA of TE cell samples was extracted, randomly fragmented, and amplified using the SurePlex DNA Amplification System (Illumina). The amplified genomic DNA from each sample was processed to prepare genomic DNA libraries using the VeriSeq PGS workflow (Illumina) at the Genetic Diagnosis Laboratory of Lee Women’s Hospital (Taichung, Taiwan). Purified DNA libraries were then normalized to equal quantities of each sample in the final pooling using the Library Normalization Additives and Beads (Illumina). The Miseq Reagent Kit v.3 (Illumina) was used on a Miseq System (Illumina). BlueFuse Multi Software (Illumina) was applied to analyze the generated bioinformatic data, and diploid-aneuploidy levels in each sample were checked by at least two technicians. Segmental gain or loss of individual chromosomes was defined as altered segments with ≥10 MB in size [16]. Based on the mosaicism levels detected with the hr-NGS platform [6,17] in biopsied TE cells, samples were classified into different groups as follows: (1) euploid blastocysts with mosaicism levels ≤ 20%; (2) low-level mosaic blastocysts with mosaicism levels > 20% and < 50%; (3) high-level mosaic blastocysts with mosaicism levels ≥ 50% and ≤ 80%; and (4) aneuploid blastocysts with mosaicism levels > 80% [1]. In addition to mosaicism levels, mosaic type (segmental, whole chromosome, and complex) and the number of chromosomes involved were analyzed in the study.

Surplus designated mosaic blastocysts from the original clinical TE biopsies were used to isolate inner cell mass as previously described to compare synchronization between single TE biopsy and ICM [18]. All ICM biopsies underwent PGT-A as described above.

## 6. Study Outcomes

Our primary outcome was live birth. Secondary outcomes included clinical pregnancy rate, ongoing pregnancy rate, multiple pregnancy rate, miscarriage rate (MR), karyotyping results, gestational age of delivery, and birth weight after SET with mosaic embryo. Implantation rate (IR) was calculated by dividing the number of gestational sacs by the total number of blastocysts transferred. Clinical pregnancy was defined as the presence of an intrauterine gestational sac with positive cardiac movement on ultrasound examination at 6–8 weeks of gestation [19]. Ongoing pregnancy rate (OPR) was defined as the number of patients with live intrauterine pregnancies beyond 12 weeks of gestation (positive cardiac activity on ultrasound examination) divided by the total number of patients who underwent embryo transfer. MR was calculated as the percentage of spontaneous intrauterine pregnancy demise confirmed by ultrasound in all clinical pregnancies [20]. Live birth rate (LBR) was defined as birth of at least one live-born child. Cumulative pregnancy rate was followed up until April 2018.

## 7. Statistical Analysis

Clinical outcome between low and high-level mosaic SET was compared using a *x*^2^ test or Fisher’s exact test for significance. A value of *p* < 0.05 was considered statistically significant. Continuous data are represented as mean ± SD and were compared between groups using the independent-samples t test. Univariable and multivariate analyses were used to examine clinical factors associated with live birth after mosaic SET. Receiver operating characteristic curves (ROCs) and measurements of area under the curve (AUC) were used to evaluate the accuracy of the predictor variables. All analyses were performed using SPSS software version 22 (IBM, Armonk, NY, USA).

## 8. Results

### 8.1. Impact of the Proportion of Chromosomal Mosaicism on Clinical Outcomes

As shown in Table 1, a total of 108 FET cycles with a single mosaic blastocyst were analyzed utilizing hr-NGS to evaluate the level of chromosomal mosaicism on clinical outcomes. Comparing CPR, IR, OPR, and LBR, no significant differences in CPR (47% vs. 52%; *p* = 0.66), IR (51.8% vs. 52%; *p* = 0.98), OPR (47% vs. 36%; *p* = 0.33), or LBR (44.6% vs. 36%; *p* = 0.45) were observed between low and high-level mosaic SETs. However, a significantly higher MR after high mosaic SETs was found (5.1% vs. 30.7%; *p* = 0.012). The miscarriage events occurred at very early gestational age before 10 weeks after high mosaic SET. Obstetric outcomes show 10.1% of multiple gestation rate in low mosaic SETs compared with no cases in the high mosaic group (*p* = 0.57) In addition, no statistically significant difference was observed in gestational weeks at delivery or in birth weight. In total, 46 women received amniocentesis for karyotyping and all results were normal. No congenital anomalies were found on prenatal ultrasound examinations.

### 8.2. Factors Affecting Live Birth in Mosaic Embryo Transfer

The clinical and laboratory factors associated with live birth in mosaic SETs were further analyzed (Table 2). Among 108 cases, there were 73 cases with segmental mosaicism, six with whole chromosome mosaicism and 29 with combined mosaicism. Univariate analysis showed that live birth correlated significantly with age of the female partner (*p* = 0.01), body mass index (BMI) (*p* = 0.04), duration of infertility (*p* = 0.07), endometrium (EM) thickness (*p* = 0.003), serum estradiol on embryo transfer (ET) day (*p* = 0.03), and endometrial preparation method (*p* = 0.003). Multivariable logistic regression analysis indicated that live birth was only correlated with age of the female partner (*p* = 0.03), BMI (*p* = 0.035) and EM thickness (*p* = 0.01) in mosaic embryo transfer. In addition, ROCs and measurements of AUC were used to evaluate the predictive capacity of age and EM thickness. The AUC for live birth was 0.72 (95% CI, 0.63–0.80), indicating an acceptable predictive ability when combining these three factors.

### 8.3. Concordance between TE Biopsy and ICM in Designated as Mosaic Embryos

Table 3 compares the concordance between the original TE biopsies and ICM. Concordance rate was 37% vs. 50% in the low and high-level groups, respectively. Although a higher rate of aneuploid ICM was observed in the high-level mosaic group, no significant difference was found in the concordance between high and low-level mosaic embryos (Supplementary File).

## 9. Discussion

This retrospective study is the first to exclusively use single mosaic embryo transfer to investigate the effects of mosaic embryos on LBR and clinical outcomes. From our early experience of 108 mosaic SET, we found that high-level mosaic embryos have comparable LBR, but higher MR compared with low-level ones. Age, BMI and EM thickness were the main factors predicting live birth in mosaic SET. Hr-NGS analysis of TE and ICM from remaining mosaic blastocysts suggests a poor concordance between ICM and TE biopsy. Blastocyst designated as mosaic blastocysts may still have an euploid ICM (high mosaicism vs. low mosaicism: 40.7% vs. 50%). Controversies remained on the effects of mosaic level on clinical outcomes. Some reports supported its relevancy, whereas others show evidence against it [7,8,13,21]. Our results may reconcile the discrepancy between them. A high discordancy between TE and ICM biopsy from embryos may result in various outcomes.

Aneuploid embryos are believed to be the main cause of IVF failure and are responsible for most implantation failures and miscarriages. Theoretically, a higher proportion of aneuploid cells within the embryos may affect its developmental potential; therefore, previous studies suggested that low-level mosaic embryos should be prioritized for transfer [1,8]. Our previous research found that the implantation and ongoing pregnancy rates in euploidy SET were significantly higher than in low-level mosaic embryo transfer groups (unpublished data) (65.7% vs. 51.8%; 64.8% vs. 47.0% *p* < 0.05). This study further showed that delivery of healthy babies was also possible even after the transfer of high-level mosaic embryos. A number of reasons may explain these results. Firstly, the self-correction hypothesis states that aneuploid cells have a growth disadvantage or are eliminated by processes such as apoptosis during embryo development [22,23,24,25]. In our previous study [12], we utilized a TL culture system to monitor the morphokinetics of mosaic embryos and found that high-level mosaic embryos exhibit features of mitotic errors and delayed development to the 8-cell stage. Bolton et al. [26] showed in a mouse model of chromosome mosaicism that aneuploid cells in ICM are eliminated by apoptosis, whereas those in TE show severe proliferative defects. Cell proliferation and death rates are elevated in mosaic blastocysts compared with euploid blastocysts, as also reported by Victor et al. [7]. The evidence shows that mosaic embryos may undergo self-correction and proceed to a state of euploid. High-level mosaic embryos may fail to self-correct owing to a higher content of aneuploid cells, resulting in abortion. Strong evidence of age as a predicting factor for live birth was also found in this study and is consistent with the self-correction hypothesis. Although previous studies have shown that advancing female age does not influence the incidence of mosaicism [17], mosaic embryos that come from young patients might undergo a better self-correction than older patients might and could be prioritized for transfer [7]. Secondly, original TE biopsies were poorly concordant with chromosomal content of ICM in mosaic embryos. The question of whether a single TE biopsy is representative of ICM remains controversial with many unsolved questions. Recently, Victor et al. reported that a TE biopsy is an excellent representative for whole chromosome aneuploidy, but not for segmental aneuploidy [18]. There are few reports on the concordance between TE and ICM in mosaic embryos. Our results reveal a high degree of inconsistency between ICM and TE in mosaic embryos. A proportion of 40% of high-level mosaic embryos still have euploid ICM and may consequently account for the live births observed. Gleicher et al. [27] also found that a single TE biopsy is mathematically unable to determine embryo ploidy accurately enough for clinical use. A 6-cell biopsy is apparently not representative of the whole TE. Insufficient TE biopsied cells may be another possible reason for the high discordance existed between TE and ICM. When analyzing a biopsy for PGS, the cellular stage also may affect the embryo diagnosis. When biopsied TE cells were in the S phase (DNA replication), more chromosomal gains or losses may be observed. Ramos et al. [28] showed that a majority of segmental imbalances result from chromosome instability during cell division. The asynchronization of TE cell division may also make the biopsied TE less representative to ICM. Therefore, caution should be exercised when discarding these embryos labeled as mosaic by TE biopsy. Finally, whether embryo mosaicism originates from natural embryonic development or is an artifact of a whole IVF or PGT-A procedure remains unknown. The incidence of mosaicism decreases as embryos progress from the cleavage stage to the blastocyst stage, but the blastocyst mosaicism rate determined using NGS methods is highly variable between laboratories, ranging from as low as 2% to as high as 40% [21,24,29]. Mosaicism related to artifacts is also a major concern. The Preimplantation Genetic Diagnosis International Society (PGDIS) stated in 2019 that the high incidence of mosaic embryos in some clinics may result from clinical treatment, laboratory manipulation, or technical effects. Previous research found that embryos via ICSI or IVF may differ in mosaicism proportion [30]. Such artifacts could arise in situations such as a poor biopsy technique with too few TE cells, cell damage, and loss of cellular DNA affecting chromosome profiles. Inadequate NGS analysis is another factor that can bias the results which may occur in library preparation, leading to incorrect copy numbers, especially for chromosomal segments [21]. BMI and EM thickness are also significant predictive factors for live birth, which are consistent with previous studies on frozen embryo transfer [31,32,33].

Furthermore, we show that an early MR is significantly higher in the high-level mosaic group, and it is necessary to consider these results when counseling patients who want to decrease the abortion rate. However, our results are inconsistent with those of Spinella et al., who reported that the extent of mosaicism influences the IVF success rate. With regard to mosaic type or number of chromosomes involved, no correlation with live birth was observed in our study. In addition, no interaction among the individual factors of mosaicism including mosaic type, extent, and number of chromosomes involved was observed. This finding is inconsistent with previous studies that suggested that complex mosaic blastocysts have a lower pregnancy rate [6]. This discrepancy can be attributed to different incidence rates of mosaicism between laboratories and the different platforms used for PGT-A. In addition, a low concordant rate between ICM and TE biopsy observed in our study may explain the different results.

ICM biopsies for PGT-A are ethically inaccessible in clinical practice, and therefore TE biopsies are a viable alternative. However, the reliability of a TE biopsy to evaluate ICM is highly controversial and makes the clinical interpretation difficult. Moreover, the mosaicism rate varies considerably among laboratories, and no appropriate techniques to differentiate true mosaicism from artifacts during the whole procedure have been proposed. Instead, individual investigations of the concordance of ICM and TE biopsy among different laboratories could offer more precise information to patients when counseling regarding mosaic embryos transfer. Although PGT-A has been shown to reduce the number of transferred embryos and to confer a higher LBR per transfer [34], the increasing use of PGT-A leads to less embryos being available for transfer owing to loss of embryos during blastocyst culture, laboratory manipulation, cryoinjury, and embryos labeled as mosaic. Caution should therefore be exercised in applying this technique routinely in IVF. Also, mosaic embryos should be reconsidered for transfer and not be classified as “abnormal”.

This retrospective study analyzed single-center data and has some limitations. First, the current study is based on a small sample of participants. Secondly, a retrospective study model may have a selection bias in which patients who prefer mosaic embryo transfer mostly had repeat IVF failure or advanced maternal age. Hence, the results may not represent all the patients undergoing the PGT-A. Randomized studies with a large sample size must be performed before mosaic embryo transfer for routine practice in PGT-A program. Furthermore, long-term follow-up data after birth of the children is still lacking. Recently, true fetal mosaicism following the transfer of a known mosaic embryo was first reported and prenatal diagnosis by amniocentesis is necessary [35].

## 10. Conclusions

The findings of this study are valuable for understanding the clinical results after SET with low/high level mosaic embryos. The present study demonstrates that high-level mosaic embryo transfer resulted in a comparable LBR, but higher MR compared with low-level mosaic embryos. The current data also found low concordance between TE biopsy and ICM in mosaic embryos and proposed reasons for why embryos designated as high-mosaic blastocysts can result in live birth. Our study suggests that mosaic embryos after PGT-A should not be discarded and should be eligible for transfer under certain circumstances. The current data allowed the evaluation of the concordance between TE biopsy and ICM, and reasons are proposed for why blastocysts designated as high mosaic can result in live births. Further research should also be conducted to investigate the differences in concordance between laboratories using NGS-based PGT-A to provide more information before the transfer of mosaic embryos.

## Figures and Tables

**Table 1:** Impact of mosaicism level on clinical outcomes after single mosaic embryo transfer.

	Low-Level Mosaic SET	High-Level Mosaic SET	*p* Value
No. of cycles	83	25	
Age of female partners (y)	36.9 ± 5.6	36.2 ± 4.8	0.38
No. of oocytes retrieved	16.4 ± 11	15.6 ± 10.8	0.88
No. of good-quality blastocysts	4.9 ± 4.7	2.62 ± 4.4	0.2
Blastocyst formation rate (%)	50.4 ± 27	31.5 ± 22	0.1
Implantation rate, (%) (n)	51.8% (43/83)	52% (13/25)	0.98
Clinical pregnancy rate, % (n)	47% (39/83)	52% (13/25)	0.66
Ongoing pregnancy rate, % (n)	47% (39/83)	36% (9/25)	0.33
Miscarriage rate, % (n)	5.1% (2/39)	30.7% (4/13)	0.012 ^a^
Live birth rate, % (n)	44.6% (37/83)	36% (9/25)	0.45
Multiple gestation, % (n)	10.8% (4/37)	0% (0/9)	0.57
Gestational age at delivery (weeks)	38.3 ± 1.6	38.6 ± 1.2	0.53
Birth weight (gm)	3015 ± 507	3076 ± 94	0.73

Note: Values are % (*n*) or mean ± SD; ^a^ *p* < 0.05.

**Table 2:** Univariate and multivariable logistic regression analyses of factors affecting live birth rate after single mosaic embryo transfer.

	Univariable Analysis		Multivariate Analysis	
Variable	OR (95% CI)	*p* Value	OR (95% CI)	*p* Value
Age, years	0.91 (0.84–0.98)	0.01 ^a^	0.89 (0.82–0.97)	0.01 ^a^
EM thickness (mm)	1.51 (1.11–2.05)	0.008 ^a^	1.54 (1.12–2.11)	0.007 ^a^
BMI (kg/m^2^)	0.86 (0.74–0.99)	0.04 ^a^	0.85 (0.73–0.99)	0.035 ^a^
Estradiol (pg/mL) on ET day	1.00 (1.00–1.02)	0.03 ^a^	1.0 (0.99–1.03)	0.06
EM preparation Nature cycle hormone replacement	0.30 (0.13–0.66)	0.003 ^a^	0.41 (0.16–1.03)	0.06
Duration of infertility (years)	0.87 (0.75–1.01)	0.07	0.90 (0.78–1.06)	0.22
No. of oocytes retrieved	0.95 (0.88–1.02)	0.19		
No. of 2PN	0.94 (0.83–1.07)			
No. of good–quality blastocysts	1.19 (0.91–1.56)	0.20		
Blastocyst rate (%)	0.22 (0.01–5.37)	0.35		
Day 5/6 blastocyst	2.2 (0.87–5.61)	0.1		
Progesterone (pg/mL) on ET day	1.0 (0.99–1.02))	0.49		
Mosaic level Low (<50%) High (≥50%)	0.67 (0.26–1.68)	0.39		
Mosaic type Segmental Whole chromosome Complex	1.75 (0.81–3.78)	0.15		
Mosaic No.	0.94 (0.77–1.15)	0.57		
Interaction				
Mosaic level x type	0.54 (0.30–0.99)	0.045 ^a^	1.74 (0.16–18.43)	0.65
Mosaic level x No.	0.90 (0.75–1.09)	0.29		
Mosaic type x No.	1.02 (0.92–1.33)	0.13		
Mosaic level x type x No.	0.89 (0.78–1.03)	0.11		

Factors with significant ORs in univariate analysis were included in multivariate analysis; statistical significance when ^a^ *p* < 0.05. OR: odd ratio; CI: confidence interval; EM: endometrium; BMI: body mass index; ET: embryo transfer; and 2PN: 2 pronuclear. Every three variables were examined at a time concerning of small sample size.

**Table 3:** Concordance between original trophectoderm (TE) biopsy and inner cell mass (ICM).

Origin TE Biopsy	ICM Results		Proportion	Concordance
**High-level mosaicism** **(N = 27)**	**Aneuploid**	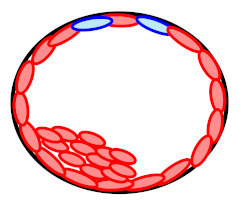	**22.2%** **(6/27)**	**Discordance**
	**Mosaicism**	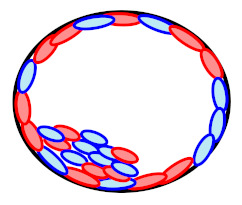	**37.0%** **(10/27)**	**Concordance**
	**Euploid**	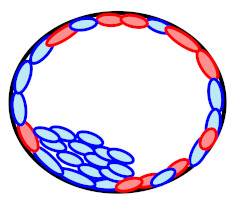	**40.7%** **(11/27)**	**Discordance**
**Low-level mosaicism** **(N = 14)**	**Aneuploid**	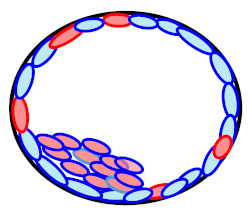	**0%** **(0/14)**	**Discordance**
	**Mosaicism**	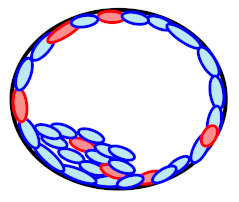	**50%** **(7/14)**	**Concordance**
	**Euploid**	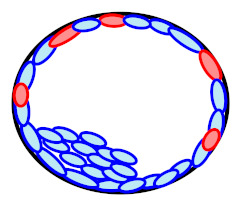	**50%** **(7/14)**	**Discordance**

## Supplementary Materials

The following are available online at https://www.mdpi.com/2077-0383/9/6/1695/s1.
