# Supplementary material for: Clinical Outcomes of Single Mosaic Embryo Transfer: High-Level or Low-Level Mosaic Embryo, Does It Matter?
Source: J Clin Med. 2020 Jun 2;9(6):1695. doi: 10.3390/jcm9061695 (PMC7356018; doi:10.3390/jcm9061695)
Supplement: Supplementary file 1 [file jcm-09-01695-s001.pdf]

Lee women hospital

胚胎品質	TE-1 Type	TE Result	ICM Type	ICM Result
BDBB	Mosaicism-Whole	Mosaicism, XX. (60% mosaic loss of chr5)	Mosaicism-Whole	Mosaicism, XX. (40% mosaic gain of chr5)
BHBA	Mosaicism-Whole	Mosaicism, XX. (80% mosaic gain of chr15)	Mosaicism-Segmental	Mosaicism, XX. (30% mosaic gain of 19p13.3~12)
BHBB	Mosaicism-Whole	Mosaicism, XX. (80% mosaic gain of chr22 ; 30% mosaic gain of 3p14.2~q11.1)	Mosaicism-Whole	Mosaicism, XX. (80% mosaic gain of chr22)
BDBB	Mosaicism-Whole	Mosacism, XX. (70% mosaic gain of chr22)	Aneuploidy-Whole	Aneuploidy, XX, +22. (70% mosaic loss of Xp22.33~11.21)
BHBC	Mosaicism-Whole	Mosaicism, XX. (80% mosaic loss of chr20 ; 30% mosaic gain of 9q12~22.1)	Aneuploidy-Whole	Abnormal, XX, -20.
BHBB	Mosaicism-Whole	Mosaicism, XY. (80% mosaic loss of chr16)	Aneuploidy-Whole	Abnormal, XY, -16. (40% mosaic gain of 13q14.11~14.3)
BHBB	Mosaicism-Whole	Mosaicism, XX. (30% mosaic gain of chr2)	Mosaicism-Whole	Mosaicism, XX. (40% mosaic gain of chr2 ; 30% mosaic gain of 19p13.3~13.11; 30% mosaic loss of 14q32.12~32.33(29%), Xq26.3~28(28%), Xq21.1~21.33)
BHBB	Mosaicism-Whole	Mosaicism, XY. (70% mosaic loss of chr22; 40% mosaic gain of 17q24.2~25.3; 40% mosaic loss of 3p26.3~24.3; 30% mosaic loss of chr2)	Aneuploidy-Whole	Aneuploidy, XY, -22.
BHBB	Mosaicism-Whole	Mosaicism, XX. (40% mosaic loss of chr5)	Mosaicism-Segmental	Mosaicism, XX. (40% mosaic gain of 3q13.12~29)
BHBB	Mosaicism-Whole	Mosaicism, XX. (60% mosaic loss of chr15)	Mosaicism-Segmental	Mosaicism, XX. (40% mosaic loss of 16p11.2~q24.3)
BHBB	Mosaicism-Whole	Mosaicism, XX. (80% mosaic loss of chr16; 30% mosaic loss of 9q22.2~22.33)	Aneuploidy-Whole	Aneuploidy, XX, -16.
BHBC	Mosaicism-Whole	Mosaicism, XX. (30% mosaic gain of chr6(26%), Xp22.33~22.2(28%))	Mosaicism-Segmental	Mosaicism, XX. (30% mosaic loss of 1q32.1~44)
BHBB	Mosaicism-Whole	Mosaicism, XX. (80% mosaic gain of chr22)	Aneuploidy-Whole	Aneuploidy, XX, +22..
BHBB	Mosaicism-Segmental	Mosaicism, XY. (80% mosaic loss of 12q14.3~24.33)	E	Euploidy, XY.
BDBB	Mosaicism-Segmental	Mosaicism, XY. (60% mosaic loss of 2p25.3~13.2; 40% mosaic loss of 6q12~14.3)	E	Euploidy, XY.
BDBA	Mosaicism-Segmental	Mosaicism, XY. (50% mosaic loss of 9q33.2~34.3; 30% mosaic gain of 4q21.23~35.1& 5p14.3~q13.3; 30% mosaic loss of 14q31.1~32.33)	E	Euploidy, XY.
BDBB	Mosaicism-Segmental	Mosaicism, XX. (poor DNA quality) (50% mosaic loss of 4q13.1~4q34.3; 40% mosaic loss of Xp22.33~11.4 & Xq13.3~28; 30% mosaic gain of 1p32.3~21.1; 30% mosaic loss of 18p11.32~q21.1)	E	Euploidy, XX.
BHBB	Mosaicism-Segmental	Mosaicism, XY. (80% mosaic loss of 5q13.2~35.3 ; 40% mosaic loss of 1q23.3~44)	Mosaicism-Segmental	Mosaicism, XY. (30% mosaic loss of 5q13.2~35.3 & 4q34.1~35.2)
BDBB	Mosaicism-Segmental	Mosacism, XY. (50% mosaic loss of 22q13.1~13.33)	Mosaicism-Segmental	Mosacism, XY. (70% mosaic loss of 22q13.1~13.33)
BDBB	Mosaicism-Segmental	Mosaicism, XX. (60% mosaic gain of 8q11.23~24.3; 50% mosaic loss of Xq21.1~21.33; 40% mosaic loss of 2p25.3~24.1)	E	Euploidy, XX.
BHCB	Mosaicism-Segmental	Mosaicism, XX. (40% mosaic loss of 16q12.2~24.3; 30% mosaic gain of chrX)	Mosaicism-Segmental	Mosaicism, XX. (40% mosaic loss of chr3 & 5p15.33~15.1; 30% mosaic gain of chr6 & 16q13~24.3)
BHBA	Mosaicism-Segmental	Mosaicism, XX. (70% mosaic loss of 7p22.3~14.3; 50% mosaic loss of 7q35~36.3; 30% mosaic loss of 7p14.2~q35; 30% mosaic gain of chr17 & chr19 & 20p13~q13.2)	E	Euploidy, XX.
BDBB	Mosaicism-Segmental	Mosaicism, XX. (40% mosaic loss of 1q41~44)	Mosaicism-Whole	Mosaicism, XX. (30% mosaic gain of chr19)
BDBB out	Mosaicism-Segmental	Mosaicism, XX. (50% mosaic loss of 13q31.1~34)	E	Euploidy, XX.
BDBC	Mosaicism-Segmental	Mosaicism, XX. (60% mosaic loss of 8p23.3~11.21 & 10q23.1~26.3; 40% mosaic gain of 6p25.3~21.2; 40% mosaic loss of 8p11.21~q24.3)	E	Euploidy, XX. (Poor DNA quality)
BDBB	Mosaicism-Segmental	Mosaicism, XY. (40% mosaic gain of 14q11.2~24.1 & 16p13.3~11.2; 40% mosaic loss of 13q31.1~34 & 17q23.2~25.3; 30% mosaic gain of 8p23.3~q24.22 & 19p13.3~q13.33 & chr22; 30% mosaic loss of 1q23.3~44 & chr6)	Mosaicism-Segmental	Mosaicism, XY.(50% mosaic loss of 15q11.2~22.2; 40% mosaic gain of 3q25.2~29 & 6q14.3~25.3 & 10q24.2~26.11; 40% mosaic loss of 8p23.3~21.2 & 16p13.3~q12.2 & chr21; 30% mosaic gain of 1q21.3~44 & 2q22.1~37.1 & 8q22.2~24.23; 30% mosaic loss of 12p13.33~11.1 & chr20) (poor DNA quality)
BDBB	Mosaicism-Segmental	Mosaicism, XX. (60% mosaic loss of 2q31.1~37.3; 40% mosaic gain of 11p11.2~q13.4 & 13q13.3~14.2; 40% mosaic loss of 2q22.1~31.1 & 2p25.3~16.3 & 11q23.3~25 & Xp22.33~21.2; 30% mosaic gain of 1p36.23~q25.3 & 4p16.1~q23 & 5p15.1~q14.1 & 12p13.33~q13.2; 30% mosaic loss of 3q26.1~29 & 6p25.3~12.3 & 6q14.1~27 & 13q22.3~34 & 14q24.3~32.33 & 16p11.2~q24.3 & Xq25~28)	E	Euploidy, XX.
BDBB	Mosaicism-Segmental	Mosaicims, XX.(30% mosaic gain of 12p12.3~11.21 & 17p11.2~q21.32)	E	Euploidy, XX.
BDCB	Mosaicism-Segmental	Mosaicims.XX.(50% mosaic loss of 7p22.3~7p11.2)	E	Euploidy, XX.
BDBB	Mosaicism-Segmental	Mosaicims, XX. (50% mosaic gain of 3p14.3~q13.31; 40% mosaic loss of 4p15.1~13, 17q11.2~23.3; 30% mosaic loss of chr7, chr11, chr13)	Mosaicism-Whole	Mosaicims, XX.(40% mosaic loss of chr10, chr20)
BHBB	Mosaicism-Segmental	Mosaicism, XX. (50% mosaic loss of 17q24.2~25.3)	Mosaicism-Segmental	Mosaicism, XX. (30% mosaic loss of 17q24.3~25.3)
BDBC	Mosaicism-Segmental	Mosaicism, XX. (30% mosaic loss of Xq12~21.33)	E	Euploidy, XX.
BHBB	Mosaicism-Segmental	Mosaicism, XX. (40% mosaic loss of Xq27.2~28)	Mosaicism-Segmental	Mosaicism, XX. (30% mosaic loss of 5p15.33~15.2)
BDBB	Mosaicism-Segmental	Mosaicism, XX. (30% mosaic gain of 5p14.1~q15, 11p12~q12.2; 30% mosaic loss of Xq21.1~28)	Mosaicism-Segmental	Mosaicism, XX. (30% mosaic loss of 4p16.3~16.1, 10q26.12~26.3)
BHBB	Mosaicism-Segmental	Mosaicism, XY. (70% mosaic loss of 7p22.3~11.2; 40% mosaic loss of 6q25.3~27; 30% mosaic gain of 20p12.3~11.23(27%))	E	Euploidy.XY.
BHAB	Mosaicism-Segmental	Mosaicism, XY. (30% mosaic gain of 5p12~q11.2, 6q22.31~23.2; 30% mosaic loss of 13q31.1~31.3)	E	Euploidy.XY.
BHBB	Mosaicism-Segmental	Mosaicism, XX. (50% mosaic loss of 17p13.3~11.1 ; 30% mosaic gain of 17q11.2~25.3(27%); 30% mosaic loss of 2p25.3~24.1(25%), 7q34~36.3)	E	Euploidy, XX.
BHBB	Mosaicism-Segmental	Mosaicism, XX. (30% mosaic loss of Xq27.3~28)	E	Euploidy, XX.
BHBC	Mosaicism-Segmental	Mosaicism, XX (30% mosaic loss of 11q24.2~25)	E	Euploidy, XX.
BHBC	Mosaicism-Segmental	Mosaicism, XX. (30% mosaic gain of 1p36.22~36.12, 2p24.1~11.1, 12q15~23.1; 30% mosaic loss of 13q33.1~34)	E	Euploidy, XX.
BHBC	Mosaicism-Segmental	Mosaicism, XY. (80% mosaic loss of 6q12~27 ; 30% mosaic loss of 8q23.1~24.3(28%), 9p21.3~13.3(26%), chrY)	Mosaicism-Segmental	Mosaicism, XY. (60% mosaic loss of 6q12~27)
